# Fixed allocation patterns, rather than plasticity, benefit recruitment and recovery from drought in seedlings of a desert shrub

**DOI:** 10.1093/aobpla/plw020

**Published:** 2016-04-12

**Authors:** Yao Zhang, Yan Li, Jiang-Bo Xie

**Affiliations:** 1 State Key Laboratory of Desert and Oasis Ecology, Xinjiang Institute of Ecology and Geography, Chinese Academy of Sciences, 818 South Beijing Road, Urumqi, Xinjiang 830011, PR China; 2 University of Chinese Academy of Sciences, 19A, Yu-Quan Road, Beijing 100039, PR China; 3 College of Life Science, Shihezi University, Shihezi 832000, PR China

**Keywords:** Allometry, biomass allocation pattern, drought, morphological adjustment, physiological regulation, recovery

## Abstract

The response of plants to drought is controlled by the interaction between physiological regulation and morphological adjustment. Although recent studies have highlighted the long-term morphological acclimatization of plants to drought, there is still debate on how plant biomass allocation patterns respond to drought. In this study, we performed a greenhouse experiment with first-year seedlings of a desert shrub in control, drought and re-water treatments, to examine their physiological and morphological traits during drought and subsequent recovery. We found that (i) biomass was preferentially allocated to roots along a fixed allometric trajectory throughout the first year of development, irrespective of the variation in water availability; and (ii) this fixed biomass allocation pattern benefited the post-drought recovery. These results suggest that, in a stressful environment, natural selection has favoured a fixed biomass allocation pattern rather than plastic responses to environmental variation. The fixed ‘preferential allocation to root’ biomass suggests that roots may play a critical role in determining the fate of this desert shrub during prolonged drought. As the major organ for resource acquisition and storage, how the root system functions during drought requires further investigation.

## Abstract (precis)

Plant morphological traits respond to drought in a rather flexible way; however, there is recent evidence of exceptions. In our study, the morphological traits of seedlings of a desert shrub were examined under drought, and we found that this species has an ‘intrinsic habit’ of investing preferentially in the roots, irrespective of drought. What is more, this inflexibility promotes its physiological recovery after drought and makes it survive in the severe desert environment. That is to say, persistence will pay off.

## Introduction

Climate change models generally agree that shifts in precipitation will result in higher drought intensity and frequency in the near future (McDowell *et al.* 2011; Jenkins and Warren 2015; Mao *et al.* 2015). Under this climate change background, increasing vegetation mortality driven by drought has been studied globally, and has triggered discussions and predictions on the responses of plants to drought (van Mantgem *et al.* 2009; Allen *et al.* 2010). The response of plants to drought is controlled by the interaction between physiological regulation (short-term scale) and morphological adjustment (long-term scale). When plants are subjected to drought stress, a series of physiological indexes (e.g. assimilative capacity, transpiration, stomatal regulation, hydraulic conductivity and respiratory rate) will change quickly (Gortan *et al.* 2009; Mengistu 2009; Xu *et al.* 2009). The physiological activity of drought-tolerant plants may be affected by even small changes in environmental water availability (Liu *et al.* 2012; Yang *et al.* 2014). In addition, recent studies have highlighted the long-term morphological acclimatization of plants to drought. For example, plants under drought develop a larger root : shoot ratio and can optimize leaf area to maintain a water supply–demand balance (Xu *et al.* 2007; Saha *et al.* 2008; Thomas *et al.* 2008). Therefore, integrated research on plants' physiological regulation and morphological adjustment will deepen our understanding of the mechanism by which plants will adapt to future climate change. However, morphological adjustment is not as well understood as physiological regulation. For example, there are still debates on how plant biomass allocation patterns respond to drought during ontogeny. Some studies showed that the biomass allocation pattern was changeable throughout the developmental stages and was dependent on water availability (Poorter and Nagel 2000; McCarthy and Enquist 2007; Wang *et al.* 2014). In contrast, recent studies argued that the biomass allocation pattern of some species showed fixed allometric trajectory with the variation of water availability (Chambel *et al.* 2007; Mao *et al.* 2012; Schall *et al.* 2012; Vilela *et al.* 2012; Xie *et al.* 2015*a*). These contradictory conclusions on plants' allometric trajectory under variation in water availability require further testing.

Early life stages, such as seedling establishment, largely determine recruitment and distribution (Song *et al*. 2013). However, the plant materials used for morphological adjustment investigations under drought were almost adult individuals, or established seedlings, with little research focussed on first-year seedlings, i.e. between germination and establishment. Recently, many studies suggest that water availability is one of the most limiting factors for seedling establishment (Narbona *et al*. 2013; Stevens *et al*. 2014). Young seedlings between germination and establishment are more likely subjected to water deficiency due to the lack of fully developed root system (Strauss and Ledig 1985; Hanson and Weltzin 2000; Tian *et al*. 2014). Hence, both morphological and physiological adjustments are key factors determining the recruitment and survival of the young seedlings during this period (Donovan and Ehleringer 1991, 1992; Mediavilla and Escudero 2010). Therefore, studies on the dynamics of the biomass allocation pattern during the seedling establishment period under drought are highly desired.

For perennial species, one of the most important strategies of drought survival is the ability to recover swiftly from drought when water becomes available again. Historically, research has concentrated on the relationships between physiological factors and recovery. For instance, hydraulic conductivity is an important factor in the recovery capacity following severe drought; however, there is a hydraulic threshold of no recovery (Lu *et al.* 2010; Meinzer and McCulloh 2013). Stomatal reopening, after drought-induced closure, leads to enhanced stomatal conductance and rapid resumption of photosynthesis throughout the recovery (Hu *et al.* 2013). A gradual decrease in osmotic pressure and a delayed increase in enzyme activities and photosynthetic efficiency have also been shown during recovery (Müeller *et al.* 2010; Luo *et al.* 2011). Recently, the contribution of morphological factors to recovery has received much attention. Morphological adjustments such as biomass allocation have been proposed as the key mechanisms used by plant species to enhance survival under disturbance (Poot and Lambers 2003; Grigg *et al.* 2010; Pichancourt and van Klinken 2012). For example, two non-phreatophyte desert species could maintain normal photosynthesis and leaf-specific apparent hydraulic conductance within a wide range of plant water status, but these physiological stabilities were based on effective morphological adjustments (Xu and Li 2006). In addition, the ‘preferential to roots’ allocation contributes to exploring more soil resources and allocating them to aboveground parts, and indicates that a large root : shoot ratio is the driver for vigorous re-growth (Drake *et al.* 2009; Pratt *et al.* 2014). A previous study also revealed that recovery and survival of Mediterranean shrubs after disturbances (i.e. fire, defoliation and drought) depend on the living roots, which provide volume for storing nutrients and water (Saura-Mas and Lloret 2014). Furthermore, these stored resources can enable sustained respiration and re-growth until the plant's photosynthesis recovers from drought to support these costs (Clarke *et al.* 2013). Unfortunately, such conclusions have mostly been drawn from experiments that only sampled once at a certain time, which may overestimate the influence of environmental variation on phenotypic plasticity (Xie *et al.* 2015*a*). A recent study, with a composite theoretical framework, proposed that the biomass allocation patterns for perennial species in stressful environments may follow a fixed allometric trajectory (Xie *et al.* 2015*a*). If biomass allocation patterns exhibit fixed allometric trajectories rather than plastic, the relationship between fixed biomass allocation patterns and recovery in stressful environments needs to be considered.

*Haloxylon ammodendron* is a widely distributed shrub in desert regions of Asia and Africa (Tobe *et al.* 2000). For *H. ammodendron*, the closely coordinated growth of root and shoot systems indicates that this shrub has a consistent biomass allocation strategy (preferential biomass allocation to belowground parts) during the course of its development (Xu *et al.* 2014; Xie *et al.* 2015*a*). However, what the biomass allocation patterns of first-year seedlings under drought would be and whether this biomass allocation pattern would contribute to recovery remains unknown. In the current study, a greenhouse experiment was performed with first-year seedlings of *H. ammodendron* grown in control, drought and re-water treatments. Physiological and morphological traits were examined throughout the drought and recovery. The basic hypotheses are that (i) the biomass may be preferentially allocated to roots along a fixed allometric trajectory throughout the first growing season from germination, irrespective of water availability; and (ii) this fixed biomass allocation pattern may be beneficial to recovery.

## Methods

### Plant material and experimental design

The experiment was performed in a greenhouse in mid-April 2013 at Fukang Station of Desert Ecology (44°17′N, 87°56′E and elevation 475 m), Chinese Academy of Sciences. The *H. ammodendron* seedlings were established from seeds in ∼10-L pots (25 cm upper diameter, 20 cm lower diameter and 25 cm height). The pots were filled with the habitat soil of *H. ammodendron* in advance—interdune sand of the southern edge of the Gurbantonggut Desert. The sand was dried and sieved before use, and the pots were watered to field capacity after being filled with sand to ensure the same initial water conditions in every pot. After germination, the plants were once supplied with 500 mL of water at 10 days (equivalent to the average soil volumetric water content in 0–2 m soil layer of the Gurbantonggut Desert in summer). The drought treatment was supplied when the seedlings had formed obvious xylem to enable measurements of leaf water potential, at ∼75 days after germination (here referred to as drought 0 day). The measurements were conducted as soon as the drought treatment was applied. When all leaves began to dry out/become friable (after ∼20 days of drought, Lu *et al.* 2010), re-watering was applied to observe recovery. Re-watering was performed at three different times (i.e. on drought 20, 30 and 40 days, respectively), and the recovered individuals were counted to calculate the recovery percentage (recovered individuals divided by total individuals). According to our preliminary experiments, 1 month is needed to confirm whether the seedlings could recover after the first-time re-watering. During this month, the frequency of re-watering was as the same as control. Physiological and morphological measurements during the recovery process were traced only on the seedlings re-watered on drought 20 days. During the experiment, plants in the greenhouse were supplied with an ambient photoperiod of 14 h day^−1^. Daily temperature was set in the range of 18–30 °C and relative humidity in the range 20–30 %.

### Treatments

The experiment consisted of three water regimes. (i) Control treatment: similar water regimes to that before treatments (500 mL 10 days^−1^). (ii) Drought treatment: conducted randomly on two-thirds of the seedlings with no further water (0 mL 10 days^−1^). (iii) Re-water treatment: half of the drought seedlings were randomly selected to have the control water regime restored (500 mL 10 days^−1^).

### Parameter measurements

Five-day periodic samplings were used to determine photosynthetic light–response curves, predawn leaf water potential (*Ψ*_pd_) and midday leaf water potential (*Ψ*_m_), shoot and root dark respiration rate, root : shoot ratio and allometric analysis of biomass allocation during drought and recovery. Eight plants were randomly selected for each trait in each treatment except for photosynthetic light–response curves (five randomly selected plants per treatment).

#### Light–response curve

The photosynthetic light–response curves were measured at 8:00–12:00 local time on sunny days using a Li-6400 portable photosynthesis system (LI-COR, Lincoln, NE). Measurements were taken on the youngest healthy leaves. Photosynthetic photon flux density (PPFD_i_) was supplied by a 20 × 30 mm leaf chamber with a red-blue light source (6400-02B), and the PPFD_i_ gradient was set at 0, 20, 50, 100, 150, 200, 400, 800, 1200, 1500, 1800, 2000 and 2200 µmol m^–2^ s^–1^. The reference carbon dioxide (CO_2_) concentration was set at 400 µmol mol^–1^, which was similar to ambient CO_2_ concentration. Gas flow rate was 500 µmol s^–1^ and chamber temperature was controlled at 30 °C. All the measurements of net photosynthetic rate (*P*_n_) on the light curves were corrected for leaf area, calculated from leaf diameters (leaves of *H. ammodendron* seedlings approximate cylinders) determined by a caliper. Regression analysis showed that the relationship between *P*_n_ and PPFD_i_ was fitted by an exponential MnMolecular function described as:

$$y=A[1-{\text{e}}^{-k(x-xc)}]$$

where *y* is net photosynthetic rate (*P*_n_), *x* is PPFD_i_, *A* is net photosynthetic rate at light saturation point, *xc* is PPFD_i_ at light compensation point and *k* × *A* is apparent quantum efficiency of photosynthesis (Xu and Li 2006).

#### Leaf water potential

Leaf water potential was determined before dawn (*Ψ*_pd_) and at solar noon (*Ψ*_m_) using a Model 3005 Pressure Chamber (PMS Instrument Company, Albany, OR). Aboveground parts were cut at the base and used for measurements, eight separate replicates for each treatment.

#### Shoot and root dark respiration rates

After the leaf water potential measurements, the aboveground parts of *H. ammodendron* seedlings were kept for dark respiration measurement and roots were rinsed free of soil with tap water (Liu *et al.* 2004; Liu and Li 2006). Both the aboveground and belowground parts were placed in a dark temperature-controlled room to equilibrate for 30 min before measurement (Reich *et al.* 2006). The CO_2_ flux was determined by a Li-840 infrared gas analyser (LI-COR) connected to the airtight dark chamber. Data were logged for 2 min (one data-point per second). The dark respiration rate (*B*, µmol s^–1^) was calculated from the slope of the linear regression between CO_2_ concentration and incubation time using the improved soil CO_2_ flux formula:

$$B=\frac{V\times P\times (1-w/1000)\times K}{R\times (T+273.15)\times 1000}$$

where *V* is the volume of the dark box (cm^3^), *P* is the atmosphere pressure (kPa), *w* is the initial water vapour fraction (mmol mol^–1^), *K* is the CO_2_-flux rate (µmol mol^–1^ s^–1^), *R* is the real gas constant (8.314 Pa m^3^ K^–1^ mol^–1^) and *T* is the initial temperature (°C) (Steduto *et al.* 2002).

All calculated dark respiration rates were finally corrected to the dark respiration rate at 27 °C by multiplication with *Q*_10_^(27 − *T*)/10^, where *Q*_10_ is the constant 1.929 (Atkin and Tjoelker 2003). The data were also transformed to the dark respiration rate per unit biomass. The measurement was repeated on eight separate replicates for each treatment.

#### Root : shoot ratio

The aboveground and belowground parts used above were dried to constant weight at 65 °C for 72 h, then biomass was weighed and root : shoot ratio was calculated.

### Statistical analysis

Data analyses were performed using SPSS 16.0 (SPSS Inc., Chicago, IL). One-way analysis of variance was used to test significance of different treatments. The water treatments were the predictor variables (treatment level was two when there were drought and control treatments only, or three when the re-water treatment was added), and the response variables were the physiological and morphological indicators mentioned above. Multiple comparison tests were performed using least significant difference method.

Standardized major axis (SMA) regression analysis (Warton *et al.* 2006) was used to compare the log_10_*Y* vs. log_10_*X* relationships (root mass vs. shoot mass, root mass vs. plant mass, shoot mass vs. plant mass and root : shoot ratio vs. plant mass) as well as the slope and intercept differences among the treatments. Calculations were carried out with the Standardized Major Axis Estimation and Testing Routines (smatr) package of R (R version 3.1.2, R Foundation for Statistical Computing). Charting was done by Origin 8.5 (Origin Lab Corp., Northampton, MA) and R 3.1.2.

## Results

### Leaf water potential

**Figure 1.**
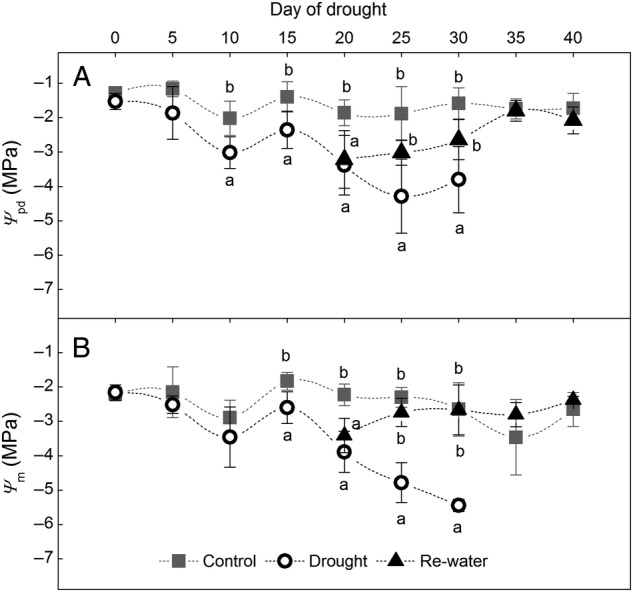
Variations of predawn leaf potential (*Ψ*_pd_) (A) and midday leaf potential (*Ψ*_m_) (B) under control, drought and re-watered conditions. Data are means ± SD; *n* = 8. Lower case letters indicate groups that are significantly different from each other (*P*< 0.05).

As the drought treatment progressed, *Ψ*_pd_ and *Ψ*_m_ of *H. ammodendron* seedlings decreased from –1.5 to –3.8 MPa and from –2.2 to –5.5 MPa, respectively (Fig. 1A and B). At 25 days of drought, the lowest *Ψ*_pd_ value of –4.3 MPa appeared (*F*_2,21_= 10.87, *P*< 0.01). For *Ψ*_m_, the lowest value of –5.5 MPa was at 30 days (*F*_2,21_= 29.71, *P*< 0.01).

After re-watering, both *Ψ*_pd_ (*F*_2,21_= 10.87, *P*< 0.01) and *Ψ*_m_ (*F*_2,21_= 40.66, *P*< 0.01) were restored to control levels. The determination of leaf water potential was not possible after drought of 30 days, because *H. ammodendron* seedlings were so withered and fragile that they broke easily when pressurized in the pressure chamber.

### Light curve

**Figure 2.**
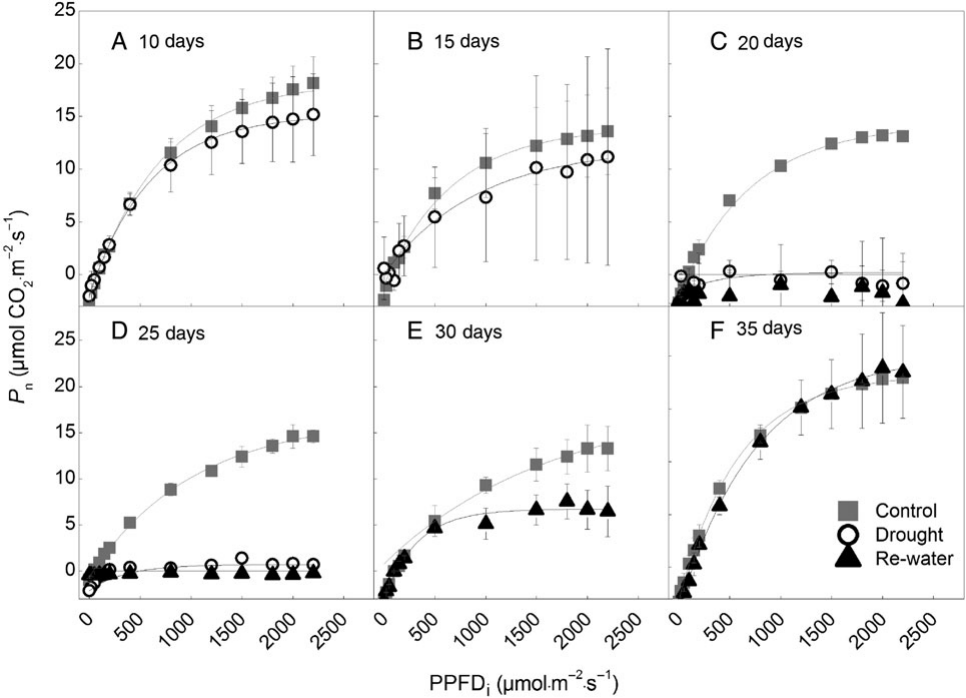
Variations of light curves at 10 days (A), 15 days (B), 20 days (C), 25 days (D), 30 days (E) and 35 days (F) under control, drought and re-watered conditions. Data are means ± SD; *n* = 5. The relationship between net photosynthetic rate (*P*_n_) and PPFD_i_ was fitted by the exponential MnMolecular function $y=A[\text{1}\text{- }{\text{e}}^{-k(x-xc)}]$, in which *y* is net photosynthetic rate, *x* is PPFD_i_, *A* is net photosynthetic rate at light saturation point, *xc* is PPFD_i_ at light compensation point and *k* × *A* is apparent quantum efficiency of photosynthesis.

The light response curves showed that the photosynthetic capacity was significantly influenced by drought (Fig. 2A–F). At 20 days of drought, the photosynthetic capacity decreased to zero (Fig. 2C), as all leaves had turned yellow. However, re-watering resulted in the re-sprouting of new leaves and improved growth. Photosynthetic capacity returned to the control level at drought 35 days (Fig. 2F).

### Shoot and root dark respiration rate

**Figure 3.**
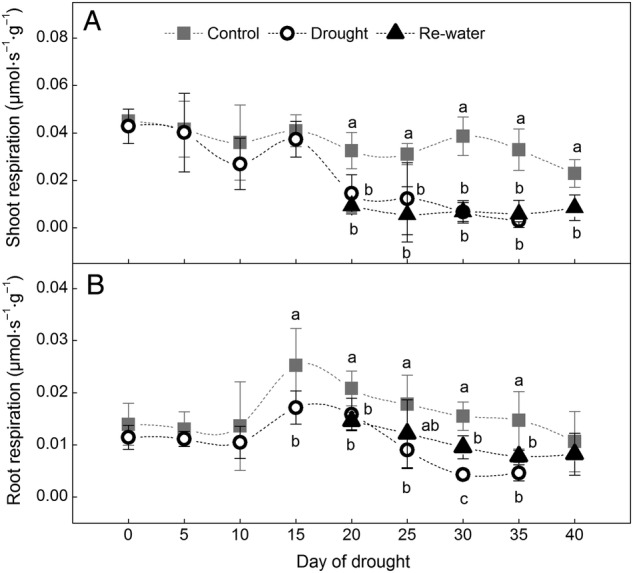
Variations of shoot (A) and root respiration (B) per unit of biomass under control, drought and re-watered conditions. Data are means ± SD; *n* = 8. Lower case letters indicate groups that are significantly different from each other (*P*< 0.05).

The shoot respiration of plants subjected to drought decreased from 4.3 × 10^–2^ to 0.3 × 10^–2^ μmol s^–1^ g^–1^ (a decrease of 93.02 %) throughout the experiment, as the whole shoot withered and turned yellow (Fig. 3A). A long time would be needed to re-grow the shoots, and shoot respiration did not recover by the end of the experiment.

Root respiration of the drought treatment gradually decreased from 1.1 × 10^–2^ to 0.5 × 10^–2^ μmol s^–1^ g^–1^ (a decrease of 54.55 %), but remained at the lower level by the end of the experiment (Fig. 3B). This showed that drought had less effect on respiration of roots compared with shoots of *H. ammodendron* seedlings. After re-watering, the root respiration returned to control levels at drought 40 days (*F*_1,14_= 0.47, *P*= 0.52; Fig. 3B).

### Root : shoot ratio and allometric relationships

**Figure 4.**
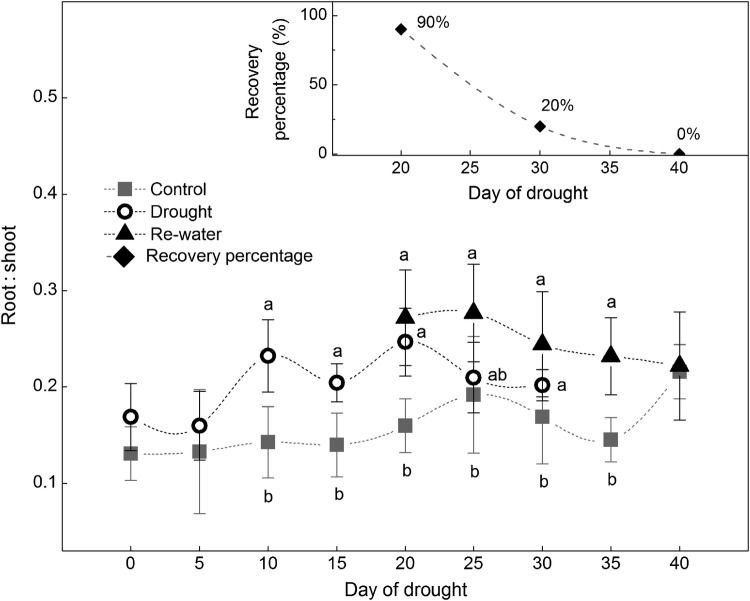
Variations of root : shoot ratio under control, drought and re-watered conditions. Data are means ± SD; *n* = 8. Lower case letters indicate groups that are significantly different from each other (*P*< 0.05). The small panel shows recovery percentages of drought seedlings on drought 20, 30 and 40 days, correspondingly.

Root : shoot ratio of drought treatment showed a significant increase at 10 days (*F*_1,12_= 17.38, *P*< 0.01), and showed an upward trend during drought (Fig. 4). However, it gradually decreased to control level after re-watering on drought 40 days. Controls also showed slight increasing root : shoot ratio throughout the experiment. Additionally, the small panel (Fig. 4) showed the recovery percentages were 90, 20 and 0 % when re-watered on drought 20, 30 and 40 days, respectively.

**Figure 5.**
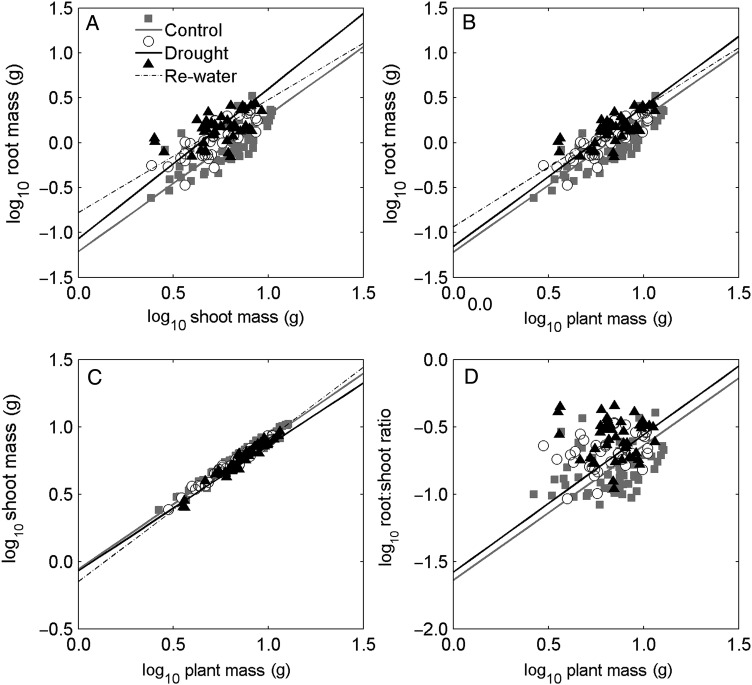
Allometric analysis for biomass allocation. Standardized major axis regression was used to test the difference in slopes and intercepts at *α* = 0.01 among treatments: log_10_ root mass vs. log_10_ shoot mass (A), log_10_ root mass vs. log_10_ plant mass (B), log_10_ shoot mass vs. log_10_ plant mass (C) and log_10_ root : shoot ratio vs. log_10_ plant mass (D). Squares and grey straight lines represent control, circles and black straight lines represent drought and triangles and black dashed lines represent re-water.

There were significant linear correlations for all treatments in the four allometric relationships, except for the re-water treatment in the relationship between root : shoot ratio and plant mass, which showed a slight negative but non-significant correlation (*R*^2^= 0.01 and *P*= 0.56, Fig. 5A–D and Table 1). The allometric relationships between root mass and shoot mass, as well as root mass and plant mass, of the *H. ammodendron* seedlings showed fixed allometric slopes (*H*_0_: slopes are equal; *P*= 0.23 and *P*= 0.50, respectively) throughout the first growth season in the three treatments (Fig. 5A and B and Table 1). In addition, the allometric relationships between shoot mass and plant mass as well as root : shoot ratio and plant mass of seedlings showed fixed allometric slopes (*P*= 0.22 and 0.90, respectively) throughout the first growth season in control and drought treatments (Fig. 5C and D and Table 1). Furthermore, there were no significant differences in intercepts between drought and control treatment in all four relationships (*H*_0_: no difference in intercepts; *P*= 0.02, *P*= 0.02, *P*= 0.03 and *P*= 0.09 for the allometric relationships of root mass vs. shoot mass, root mass vs. plant mass, shoot mass vs. plant mass and root : shoot ratio vs. plant mass, respectively).

**Table 1.** Results for SMA slopes fitted within treatments in root mass vs. shoot mass, root mass vs. plant mass, shoot mass vs. plant mass and root : shoot ratio vs. plant mass. Testing for common slopes (where slopes are equal, *P* > 0.01) and intercept differences (where no differences in intercept, *P* > 0.01). *R*, root mass; *S*, shoot mass; *M*, plant mass; *RS*, root : shoot ratio; LR, likelihood ratio statistic; *W*, wald statistic. ^1^The fitted curves were compared only between the control and drought treatments. The reason is given in the ‘Fixed biomass allocation pattern’ section.

*Y*	*X*	Slopes (99 % confidence intervals) *R*^2^, *P*			LR, df, *P*	Intercepts (99 % confidence intervals)		*W*, df, *P*
		Control	Drought	Re-water	(*H*_0_: slopes are equal)	Control	Drought	(*H*_0_: no difference in intercept)
*R*	*S*	1.52 (1.21, 1.91) 0.55, *P* < 0.01	1.67 (1.29, 2.18) 0.60, *P* < 0.01	1.26 (0.89, 1.78) 0.27, *P* < 0.01	2.94, 2, 0.23	−1.21 (−1.49, −0.94)	−1.15 (−1.49, −0.81)	5.57, 1, 0.02^1^
*R*	*M*	1.49 (1.23, 1.79) 0.70, *P* < 0.01	1.56 (1.27, 1.91) 0.76, *P* < 0.01	1.33 (0.99, 1.78) 0.48, *P* < 0.01	1.40, 2, 0.50	−1.28 (−1.52, −1.04)	−1.21 (−1.48, −0.94)	5.49, 1, 0.02^1^
*S*	*M*	0.97 (0.92, 1.03) 0.98, *P* < 0.01	0.93 (0.87, 1.01) 0.97, *P* < 0.01	1.06 (0.96, 1.16) 0.95, *P* < 0.01	1.50, 1, 0.22^1^	−0.04 (−0.09, 0.00)	−0.04 (−0.10, 0.02)	4.97, 1, 0.03^1^
*RS*	*M*	1.00 (0.72, 1.38) 0.08, *P* < 0.05	1.02 (0.71, 1.48) 0.19, *P* < 0.01	−1.20 (−1.79, −0.80) 0.01, 0.56	0.02, 1, 0.90^1^	−1.65 (−1.94, −1.37)	−1.50 (−1.83, −1.17)	2.89, 1, 0.09^1^

## Discussion

Physiological and morphological indicators in first-year *H. ammodendron* seedlings were examined during drought and recovery in order to develop an integrated understanding of the physiological regulation and morphological adjustment. We found that the biomass of *H. ammodendron* seedlings was preferentially allocated to roots, irrespective of water availability. This fixed allocation pattern contributed to recovery after re-watering.

### Physiological responses and morphological adjustment

The decreased photosynthesis (Fig. 2) and respiration (Fig. 3) denote a reduction in growth. Recent studies suggested that growth reduction can lower the growth costs and thereby ensure survival and persistence under stress (McDowell 2011; McDowell *et al.* 2011). In contrast, for Hawaiian *Metrosideros polymorpha*, photosynthetic capacity is higher at dry sites, as this would enable sustained growth across a dramatic range of water supply (Cornwell *et al.* 2007). That is, when photosynthetic capacity is limited under drought, plants will have a ‘trade-off’ between growth and survival (Eckstein 2005). Consequently, for the desert shrub *H. ammodendron*, reduced growth can lead to a less severe strain on carbohydrate availability, and finally ease the stress and increase the survival probability.

The gradual increase in root : shoot ratio (Fig. 4) during drought in the present study represents effective morphological adjustment. Similar results were also found in field experiments for the adult plants of *H. ammodendron* (Xu *et al.* 2007). Integrated research on physiological regulation and morphological adjustment also highlighted that the morphological adjustment is driven by the xylem transport capacity (Li *et al.* 2005; Plavcová and Hacke 2012; Smith *et al.* 2014; Mencuccini 2015). Furthermore, the role of morphological adjustments combined with physiological regulation is especially important for first-year seedlings, which are more likely to suffer from water deficiency compared with larger trees (Hanson and Weltzin 2000). Our study demonstrated efficient physiological regulation and morphological adjustment in first-year *H. ammodendron* seedlings subject to drought, and emphasized the integrated role of the two.

### Fixed biomass allocation pattern

The allometric relationships of the *H. ammodendron* seedlings exhibited relatively fixed allometric trajectories, supports our first hypothesis: biomass of *H. ammodendron* seedlings is preferentially allocated to roots during the first-year seedling period, irrespective of water availability. However, there is a slightly negative correlation in the allometric relationship between root : shoot ratio and plant mass in re-watered group (Table 1). Sustained drought not only results in the gradual death of the seedling root system but also limits the decomposition activity of rhizosphere microorganisms (Vivanco and Austin 2006). Thus, the decomposition rate of dead roots is low in drought due to low microbial activity. A recent study in the same region (i.e. the southern edge of the Gurbantonggut Desert) revealed that faster root decomposition rate can be due to favourable decomposition factors such as higher moisture conditions, suitable temperatures and closer contact with microbial communities in soil (Zhao *et al.* 2014). Therefore, re-watering significantly enhanced the rhizosphere microbial activity and accelerated the decomposition of dead roots, which led to a decreased root : shoot ratio and thus a slightly negative correlation.

Recent study shows that, if environmental predictability is poor, being very responsive to the environment can be detrimental (Van Buskirk and Steiner 2009; Chevin *et al*. 2013). In some sense, fixed allometric allocation patterns can decrease the costs for plasticity while increasing fitness (e.g. survival rate) in stressful environments (DeWitt *et al.* 1998; Poorter *et al.* 2012; Shemesh and Novoplansky 2013). In the Gurbantonggut Desert, the precipitation is infrequent, discrete and largely unpredictable (Xu and Li 2006; Li *et al.* 2011; Salguero-Gómez *et al*. 2012). Thus, the conservative strategy (i.e. fixed biomass allocation pattern; Xie *et al*. 2015*a*, *b*) of *H. ammodendron* could facilitate their survival and persistence throughout the first-year development. In addition, there are a number of examples showing that natural selection in stressful environments favours the evolution of fixed traits (Fortunel *et al.* 2009; Vilela *et al.* 2012).

### Relationship of fixed biomass allocation pattern and recovery

When the root : shoot ratio of the drought treatment was higher than control on drought 20 days, the recovery percentage after re-watering was 90 %, and all physiological and morphological indicators recovered to control levels in the end of the experiment except the dark respiration for shoot. After drought 20 days, the root : shoot ratio of the drought treatment began to decrease because sustained respiration and loss of photosynthetic ability resulted in smaller plant size, which moved back along the allometric trajectory. This reduction in root : shoot ratio was associated with a 20 % decrease in recovery on drought 30 days. Recovery capacity was totally lost by drought 40 days (Fig. 4). Nonetheless, from the allometric perspective, drought affected the plant size but not the allometric relationship between root : shoot ratio and plant size (i.e. fixed biomass allocation pattern; Fig. 5). This result is consistent with the literature showing that any factor that affects plant size will also affect the allocation proportion of different organs (Weiner 2004). Although the root : shoot ratio decreased after drought 20 days, it was still on the allometric trajectory (just moved back along the trajectory). Consequently, these results support our second hypothesis that a fixed biomass allocation pattern benefits recovery.

In general, species that can recover following drought have a higher allocation to roots than shoots relative to species not able to recover following drought (Zeppel *et al.* 2015). A recent study also demonstrated that a high root : shoot ratio facilitated the acquisition of soil resources and transport to aboveground tissues (i.e. nutrients and water; Saura-Mas and Lloret 2014), thus providing for enhanced growth and carbohydrate storage (Drake *et al.* 2013). A previous study on *H. ammodendron* revealed that a fixed allometric allocation pattern will decrease the cost of plasticity, while at the same time increase fitness under stress (Xie *et al.* 2015*a*). In addition, preferential biomass allocation to roots for water acquisition, and corresponding efficient morphological adjustment influences survival and persistence (Xu and Li 2006; Xu *et al.* 2007). Similar results were also found in the greenhouse experiment we report here. It is also possible that, given the infrequent, discrete and largely unpredictable precipitation in the Gurbantonggut Desert, continuous preferential biomass allocation to roots may enable *H. ammodendron* seedlings to access deeper soil layers and avoid highly saline surface soils, while at the same time access deeper water during summer drought. This fixed biomass allocation pattern could also provide access to the soil resources required for recovery during the wet season. Similar results were found for *Quercus nigra* in a pine–grassland ecosystem, in which they had a life-history strategy of maintaining belowground biomass over aboveground growth to enable persistence and recovery with frequent top-kill (Hmielowski *et al.* 2014).

## Conclusions

The current study emphasizes the effect of physiological regulation and morphological adjustment on survival and recovery for *H. ammodendron* seedlings during their first-year development. We found that (i) biomass was preferentially allocated to roots along a fixed allometric trajectory, irrespective of water availability; and (ii) this fixed biomass allocation pattern was beneficial to recovery after re-watering. An allocation pattern reflects the organism's priorities for different organs throughout its development. The fixed ‘preferential to root’ biomass allocation pattern reveals that roots may play a critical role in determining the fate of desert perennials in prolonged drought. As the major organ for resource acquisition and storage, how the root system functions during drought requires further investigation.

## Sources of Funding

Financial support was from the National Natural Science Foundation of China (grant numbers: 41371079 and 41171049).

## Contributions by the Authors

Y.L. conceived the study; Y.Z. performed research and wrote the paper; Y.Z. and J-B.X. analysed the data.

## Conflict of Interest Statement

None declared.
